# Design of HR1 and HR2 synthetic peptide analogs: Characterization of their antiviral activities and their capacities to induce neutralizing antibodies

**DOI:** 10.1186/1742-4690-10-S1-P112

**Published:** 2013-10-11

**Authors:** Olfa Mzoughi, Meritxell Teixido, Ibtissem Hamimmed, Esther Zurita, Miguel Moreno, Fabrice Gaston, Giovana Granados, Ernest Giralt, Elmostafa Bahraoui

**Affiliations:** 1 INSERM, U1043, CPTP, CHU purpan, Toulouse, France; 2 Université Paul Sabatier, 118 Route de Narbonne, Toulouse, France; 3 Institut de Recerca Biomèdica de Barcelona, Parc Cientific de Barcelona, C/Josep Samitier, Barcelona, Spain

## Background

HIV-1 entry is a dynamic process with two main actors: HIV-1 envelope glycoproteins: gp120 and gp41. The entry is a key step of the infection: blockade of this step leads to an inhibition of the viral replication. Our main focus in this study is the key actor of the viral entry and fusion: gp41. Our goal is to develop HIV-1 entry inhibitors using two strategies: i) the development of immunogens capable to induce neutralizing antibodies, ii) the modeling and synthesize as monomeric or trimeric peptide inhibitors derived from the HR1 and HR2 regions of gp41.

## Material and methods

HR1 and HR2 regions of HIV-1 gp41 were synthesized chemically as monomeric or trimeric peptides. Punctial D-amino acid substitutions (C34M2 and C34 M4) were introduced at the more susceptible enzymatic cleavage sites. These peptides were then tested for their antiviral activity and their capacity to elicit neutralizing antibodies in mice.

## Results

We studied, in the first part, the complex formed by the HR1 and HR2 domains, the purification of this complex, its stability and its ability to induce neutralizing antibodies, in a vaccine approach. We showed that HR1-HR2 complex is rapidly formed (less than 1 min) and remained stable as demonstrated by its inability, in contrast to each free peptide, to inhibit syncytia formation. Purified preformed HR1-HR2 complex and monomeric HR1 and HR2 peptides are immunogenic in mice and induce antibodies that recognize total HIV-1 envelope. They neutralize fusion when incubated at 27°C.

In a second approach trimeric synthetic peptides modelling HR1 and HR2 of HIV-1 gp41 were tested for their antiviral activities and used as immunogens in mice. The obtained results showed that these trimers could interact with the HR1 or HR2 regions and had a similar ,for the trimerC34, and an ameliorated antiviral activity, for the TrimerN36, compared to the monomer C34 and N36. More interestingly we showed that antibodies produced against trimeric HR1 and HR2 or against the hexameric complex HR1-HR2 were able to recognize native envelope glycoproteins gp41 and gp160 of HIV-1 and to inhibit syncytia formation in co-culture of HeLa cells expressing CD4-CC5R/CXCR4 receptors and HeLa cells stably expressing gp120/gp41 from R5 or X4 tropic viruses.

## Conclusions

Our data showed that: -the introduction of punctual D-amino acid substitutions can help to increase the stability of the peptides without altering their antiviral activity, - HRI and HRII, modelled as trimeric peptides, conserve their antiviral activities and are able to induce anti-peptide antibodies which recognize native gp41 and gp160 envelope glycoproteins and interfere with cell-cell fusions at physiological conditions.

